# Emerging Roles of Vascular Cell Adhesion Molecule-1 (VCAM-1) in Immunological Disorders and Cancer

**DOI:** 10.3390/ijms19041057

**Published:** 2018-04-02

**Authors:** Deok-Hoon Kong, Young Kwan Kim, Mi Ra Kim, Ji Hye Jang, Sukmook Lee

**Affiliations:** Research Center, Scripps Korea Antibody Institute, Chuncheon 200-701, Korea; kong0131@skai.or.kr (D.-H.K.); youngk@skai.or.kr (Y.K.K.); cslove526@skai.or.kr (M.R.K.); jjh717@skai.or.kr (J.H.J.)

**Keywords:** antibody, cancer, inflammation, immunological disorder, therapeutic target, tumor necrosis factor α, vascular cell adhesion molecule-1

## Abstract

Tumor necrosis factor alpha (TNFα) is a pro-inflammatory cytokine that triggers the expression of inflammatory molecules, including other cytokines and cell adhesion molecules. TNFα induces the expression of intercellular cell adhesion molecule-1 and vascular cell adhesion molecule-1 (VCAM-1). VCAM-1 was originally identified as a cell adhesion molecule that helps regulate inflammation-associated vascular adhesion and the transendothelial migration of leukocytes, such as macrophages and T cells. Recent evidence suggests that VCAM-1 is closely associated with the progression of various immunological disorders, including rheumatoid arthritis, asthma, transplant rejection, and cancer. This review covers the role and relevance of VCAM-1 in inflammation, and also highlights the emerging potential of VCAM-1 as a novel therapeutic target in immunological disorders and cancer.

## 1. Introduction

Tumor necrosis factor alpha (TNFα) is a member of the TNF ligand superfamily, which are primarily produced by immune cells, including macrophages, T lymphocytes, and natural killer cells [1]. TNFα helps regulate immunologic, hematopoietic, and pro-inflammatory activities [2,3]. In 1975, TNFα was first isolated by Carswell et al. from the sera of mice infected with Bacillus Calmette-Guérin and was identified as a TNF in Meth A sarcoma cells and other transplanted tumors [4]. The matrix metalloprotease TNFα-converting enzyme processes TNFα into 157 amino acid residues (17 kDa) via proteolytic cleavage between residues alanine 76 and valine 77 [5]. This soluble form of TNFα specifically binds to TNF receptor 1 (TNFR1), a type I transmembrane protein, which is expressed in almost all cells as pre-assembled trimers [5,6,7]. Structurally, the extracellular components of TNFR1 comprise three well-ordered cysteine-rich domains (CRD1, CRD2, and CRD3), and a less conserved fourth CRD [8,9]. Among the CRDs, CRD2, and CRD3 are mainly involved in TNFα binding [10]. The intracellular domain of TNFR1 contains a death domain (DD) [10].

The binding of TNFα to TNFR leads to receptor homotrimerization and the recruitment of adaptor proteins to the intracellular domain, resulting in inflammation, apoptosis, reactive oxygen species (ROS) generation, cell proliferation, and cell survival [11,12]. These pleiotropic bioactivities of TNFα are associated with diseases, including diabetes, heart failure, atherosclerosis, cancer, sepsis, and autoimmune diseases [1,13,14,15,16,17]. Upon binding to TNFα, TNFR1 induces several intracellular signaling pathways, including nuclear factor κB (NF-κB) and mitogen-activated kinase (MAPK) pathways [11,12]. The DD of the intracellular tail of TNFR1 rapidly recruits TNFR1-associated death domain protein (TRADD) and complexes with TNF receptor-associated factor 2 (TRAF2), receptor-interacting protein 1 (RIP1), and cellular inhibitor of apoptosis proteins (cIAP1/2) [12,18]. Subsequently, these complexes stimulate the transforming growth factor-β-activated kinase 1 (TAK1) signaling complex, which is composed of TAK1, TAK1 binding proteins 2 and 3 (TAB2 and TAB3), and the inhibitor of κB (IκB) kinase (IKK) signaling complex, which includes the NF-κB essential modulator (NEMO) and IKK subunits α and β, through the scaffolding ubiquitin network [12,18]. The TAK1 signaling pathway in turn triggers MAPK signaling cascades, leading to c-jun N-terminal kinase (JNK), P38, and AP1 activation, whereas the IKK signaling complex activates the NF-κB pathway via the phosphorylation of IκBα [12,18]. Subsequently, TNFR1 signaling induces the expression of NF-κB and AP1 target genes, including *E-selectin*, intracellular adhesion molecule-1 (*ICAM-1*), and vascular cell adhesion molecule-1 (*VCAM-1*) [11,12,18,19].

VCAM-1 (CD106) is a 90-kDa glycoprotein that is inducible and predominantly expressed in endothelial cells. In 1989, VCAM-1 was first identified as an endothelial cell surface glycoprotein [20,21]. VCAM-1 expression is activated by pro-inflammatory cytokines, including TNFα, and also by ROS, oxidized low density lipoprotein, high glucose concentration, toll-like receptor agonists, and shear stress [22]. Under high levels of inflammation and chronic conditions in some diseases, VCAM-1 also is expressed on the surface of other cells, including tissue macrophages, dendritic cells, bone marrow fibroblasts, myoblasts, oocytes, Kupffer cells, Sertoli cells, and cancer cells [23,24]. Structurally, human VCAM-1 contains an extracellular domain with six or seven immunoglobulin (Ig)-like domains, a transmembrane domain, and a cytoplasmic domain, whereas the mouse VCAM-1 has a three or seven Ig-like domain form [22,25]. The Ig-like domains of the extracellular domain contain both the disulfide-linked loops and the *N*-glycosylation site that binds to galectin-3 on eosinophil [22,25,26]. In addition to galectin-3, Ig-like domain 1 and/or 4 of VCAM-1 is involved in ligand binding, including α4β1 integrin and α4β7 integrin [22,25]. α4β1 integrin plays a major role in the VCAM-1–mediated rolling and firm adhesion of leukocytes to the endothelium, as well as leukocyte transmigration [27,28].

**Figure 1:**
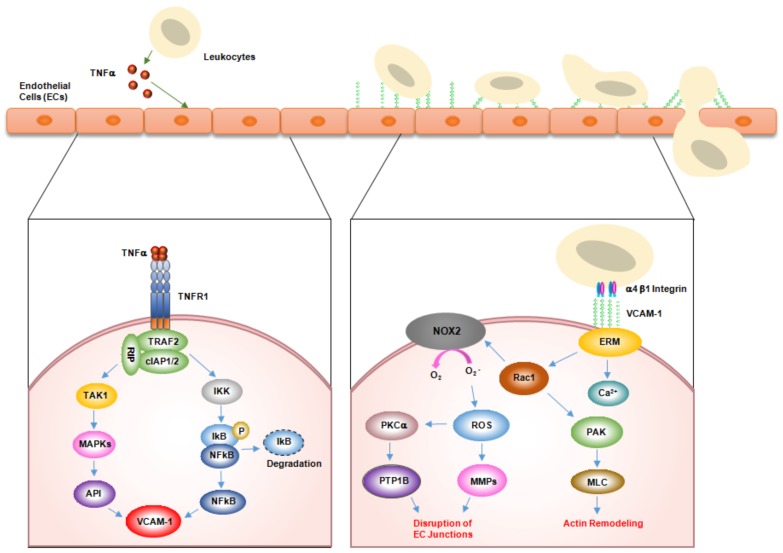
Mechanism of vascular cell adhesion molecule-1 (VCAM-1)–mediated leukocyte adhesion and transendothelial migration across endothelial cells. In inflammation, tumor necrosis factor alpha (TNFα, which is mainly secreted from leukocytes, upregulates VCAM-1 expression on the surface of endothelial cells. VCAM-1 on activated endothelial cells directly interacts with α4β1 integrin on leukocytes. In turn, this interaction activates VCAM-1 downstream signaling molecules, including Ca^2+^, Rac1, nicotinamide adenine dinucleotide phosphate oxidase 2 (NOX2), reactive oxygen species (ROS), metalloproteinases (MMPs), protein kinase Ca (PKCα), and protein tyrosine phosphatase 1B (PTP1B). Eventually, these signals relax the affinity of junction adhesion molecules within endothelial cell inunctions, allowing leukocytes to migrate through the junction.

During inflammatory responses, ligands binding to VCAM-1 on the surface of activated endothelial cells first initiate the activation of calcium fluxes and Rac1 [22,29]. In turn, the calcium fluxes and Rac1 induce the downstream activation of nicotinamide adenine dinucleotide phosphate (NADPH) oxidase 2, leading to ROS generation [29]. NADPH oxidase produces superoxide from oxygen using the cofactor NADPH, followed by dismutation to hydrogen peroxide (H_2_O_2_). This intracellular H_2_O_2_ markedly affects signal transduction and leads to the activation of matrix metalloproteinases and protein kinase Cα (PKCα) [30,31]. Activated PKCα in turn increases the serine phosphorylation of the protein tyrosine phosphatase 1B (PTP1B) on the endoplasmic reticulum, which activates PTP1B. PTP1B activation is required for VCAM-1–dependent leukocyte transendothelial migration [31,32]. VCAM-1 also stimulates the formation of actin stress fibers via the Rac1-p21-activated protein kinase-myosin light chain signaling pathway [31]. Finally, this signal transduction pathway leads to gap formation or junctional weakening of endothelial cell–cell interaction that facilitates leukocyte transendothelial migration under inflammation conditions [22,31] (Figure 1).

## 2. Role of VCAM-1 in Inflammation

Inflammation is a protective biological response that recruits immune cells, blood vessels, and molecular mediators to eliminate harmful stimuli, including bacteria, viruses, or damaged cells. In inflammation, leukocyte trafficking is regulated by the complicated and coordinated actions of many molecular mediators, including chemokines, selectins, and cell adhesion molecules [33,34]. Generally, inflammation is initiated by the release of TNFα from immune cells, such as macrophages, T lymphocytes, and natural killer cells [35]. In turn, TNFα triggers a series of various cell adhesion molecules, such as selectins, ICAM-1, and VCAM-1, to recruit a subset of leukocytes at inflamed sites through leukocyte adhesion [19]. Among these adhesion molecules, VCAM-1 is a major regulator of leukocyte adhesion and transendothelial migration through interaction with α4β1 integrin. α4β1 integrin expressed on leukocytes adheres to VCAM-1 on the surface of endothelial cells, and activates signaling pathways within the activated endothelial cells that allow the transendothelial migration of leukocytes [28]. VCAM-1 and α4β1 integrin play a central role in leukocyte recruitment during inflammation. In 1989, Osborn et al. reported that Jurkat T cells and Ramos cells had more adhesion to TNFα-treated human umbilical vein endothelial cells (HUVECs) and VCAM-1–transfected COS-7 cells than untreated HUVECs and control vector-transfected COS-7 cells, respectively [21]. This finding suggested that VCAM-1 is critical for the adhesion of lymphocytes to human endothelial cells [21]. Additionally, in follicular dendritic cells, α4β1 integrin was identified as a VCAM-1 ligand that is required for the firm adhesion of B cells to lymphoid germinal centers [36]. In other studies, α4β1 integrin-transfected K562 erythroleukemia cells exhibited tethering and rolling in a VCAM-1-coated flow chamber with TNFα-stimulated HUVEC monolayers, and the siRNA-mediated knockdown of VCAM-1 in subcutaneous hemangioma endothelial cells (sEnd1) reduced the adhesion of Jurkat T cells to monolayers of sEnd1, demonstrating the importance of the interaction between α4β1 integrin and VCAM-1 in inflammation [27,37]. Further, in dextran sulfate sodium-induced colitis mouse models, the neutralization of VCAM-1 by anti-VCAM-1 antibody (MK1.91) disabled leukocyte adhesion to the endothelium and significantly attenuated colitis [38]. In mouse models of ovalbumin (OVA)-induced pulmonary inflammation, the recruitment of mast cell precursors was significantly lower in VCAM-1 knockout mice than in wild-type mice [39]. In the same model, the administration of an anti-VCAM-1 antibody (429) also had reduced the recruitment of mast cell precursors to the inflamed lung [39]. Furthermore, the anti-VCAM-1 antibody attenuated macrophage, neutrophil, and eosinophil recruitment in an OVA-induced murine allergic asthma model [40]. 

To understand the role of VCAM-1 in inflammation, it is important to understand the specific roles of each VCAM-1 domain. The extracellular domain of VCAM-1 contains seven Ig-like domains [22]. Ig-like domain pairs of 1 and 4, 2 and 5, and 3 and 6 are highly homologous with each other [22]. Domain 1 (and/or 4) of VCAM-1 is involved in the direct binding of α4β1 integrin, resulting in leukocyte adhesion [22]. Osborn et al. showed that treatment with anti-VCAM-1 domains 1 or 4-blocking antibodies (4B9 and ED11) specifically reduced the interaction of α4β1 integrin-expressing Ramos cells with TNFα-stimulated HUVECs and VCAM-1-overexpressing CV-1 (simian) in Origin, and carrying the SV40 genetic material (COS) cells in vitro [41]. Additionally, Q38S, D40A, and L43NAD mutation at domain 1 or D328A and L331A mutation at domain 4 significantly inhibited the binding of Ramos cells to VCAM-1-overexpressing COS cells [42]. In addition to the functions of Ig-like domains 1 and 4, we recently developed a rabbit/human chimeric monoclonal antibody specific to the Ig-like domain 6 of VCAM-1 (anti-VCAM-1-D6 chimeric mAb) [43]. Using this antibody, we demonstrated that Ig-like domain 6 is important for leukocyte transmigration, but not for leukocyte adhesion [43]. The VCAM-1-D6 antibody specifically blocked the transmigration of U937 human promonocytic leukocyte cells through TNFα-stimulated HUVECs, but did not affect the adhesion of U937 cells to TNFα-stimulated HUVECs [43]. Together, these data suggest that the Ig-like domains 1 and 4 of VCAM-1 play a crucial role in the binding of the α4β1 ligand for leukocyte adhesion to the endothelium, whereas Ig-like domain 6 enables leukocyte transmigration in inflammation.

## 3. Role of VCAM-1 in Immunological Disorders

### 3.1. Rheumatoid Arthritis (RA)

Rheumatoid arthritis (RA) is a systemic and chronic autoimmune disease that is characterized by symmetric polyarticular joint disorders that primarily affect the small joints [44,45]. Although various factors are related to the pathogenesis of RA, TNFα predominantly mediates inflammation, ultimately leading to joint deformation, destruction, and disability [46,47]. In RA, leukocytes travel to the joint and produce cytokines including TNFα. Then, these proteins stimulate the cells to attack healthy tissues, leading to inflammation and the progressive damage of cartilage, bone, and other joint-related tissues [44,45,47]. To date, there are three TNFα inhibitors that have been approved by the United States Food and Drug Administration [48,49,50]. Infliximab, a chimeric monoclonal antibody, neutralizes soluble and transmembrane TNFα, and prevents TNFα from binding to its receptor [48]. Etanercept, a fusion protein with the extracellular portion of TNFα receptor and the Fc region of human IgG1 antibody, targets soluble TNFα [49]. Adalimumab, a humanized monoclonal antibody targeting TNFα, also neutralizes both soluble and transmembrane TNFα [50].

VCAM-1 expression is closely associated with RA. Wang et al. showed that serum VCAM-1 levels were much higher in patients with RA than in controls, and that the prolonged use of aspirin, a non-steroid anti-inflammatory drug, decreased serum levels of rheumatoid factor and VCAM-1, suggesting that serum levels of VCAM-1 may be related to the disease condition [51]. Klimiuk et al. also demonstrated high levels of serum VCAM-1 in RA patients with follicular synovitis [52]. Smith et al. investigated the effect of disease-modifying anti-rheumatic drugs (DMARDs) such as methotrexate and intramuscular gold on RA patients [53]. They observed dramatically lower VCAM-1 levels in the DMARDs-treated group [53]. Oberoi et al. also showed that adalimumab, a leading RA therapeutic antibody, suppresses the upregulation of VCAM-1 mRNA and protein expression in HUVECs activated by TNFα, further suggesting the close relationship between VCAM-1 and RA [54].

The interaction between VCAM-1 and α4β1 integrin seems to be critical for RA. Carter et al. investigated the role of VCAM-1 in an autoimmune mouse model of RA by treating mice with collagen-induced arthritis (CIA) with a neutralizing monoclonal antibody (M/K-2.7) [55]. They found that the antibody significantly reduced the overall clinical severity of the disease in comparison with a control antibody [55]. Using histological analyses, they also observed fewer arthritic joints in the paws of M/K-2.7–treated mice [55]. Morales-Ducret et al. also reported the inhibition of binding between Jurkat cells and resting fibroblast-like synoviocytes (FLS) monolayers that were exposed to an antibody to α4β1 integrin (VLA-4), a CS-1 peptide from an alternatively spliced fibronectin (which is another VLA-4 ligand), and, to a lesser extent, an anti-VCAM-1 antibody [56]. In addition, B lymphocytes accumulated in the inflamed joints of patients with RA and produced high amounts of (auto)antibodies [56]. Reparon-Schuijt et al. showed that synovial fluid B cells undergo spontaneous cell death by apoptosis and are rescued by interactions with FLS [57]. Further, they demonstrated that an anti-VCAM-1 blocking antibody (1.G11B1) reduced the survival of synovial fluid B cells by inhibiting their interaction with FLS, further supporting the close relationship of α4β1 integrin and VCAM-1 in RA [57]. Furthermore, Shimada et al. demonstrated that interleukin-4 (IL-4) stimulated the expression of VCAM-1, but not ICAM-1, on synovial cells, and the IL-4-stimulated synovial cells had increased adhesion to T cells, which is mediated by the binding of α4β1 integrin to VCAM-1 [58]. They also showed that the adhesion of T cells to synovial cells was inhibited by a murine anti-VCAM-1 monoclonal antibody (BBA6) or anti-α4β1 integrin (HP2/1), implying that the interaction between VCAM-1 and α4β1 integrin may play an important role in RA synovium [58]. Silverman et al. also showed the accumulation of endothelial progenitor cell in synovium in RA using the CIA mouse model, which suggests that the interaction of VCAM-1 with α4β1 integrin mediates the recruitment of endothelial progenitor cells to promote neovascularization in RA synovial cells [59].

### 3.2. Asthma

Asthma is a chronic inflammatory disorder of the airways that is associated with bronchial hyperreactivity, reversible airflow obstruction, and bronchospasm [60,61,62,63,64]. In the asthmatic lung, IL-4 promotes VCAM-1 expression, leading to the VCAM-1-mediated adhesion of eosinophils to activated endothelium [65,66,67,68]. In turn, the adhesion of blood eosinophils to VCAM-1 further induces the transmigration of eosinophils across activated endothelial cells, resulting in respiratory burst and the enhanced release of granule proteins in inflamed tissues [69,70,71].

Several studies have shown that VCAM-1 expression is critical for eosinophil infiltration in asthma [72,73,74]. Ohkawara et al. reported that endothelial VCAM-1 expression in bronchial mucosa from patients with asthma correlates with eosinophil migration into the airways [72]. Fukuda et al. showed the increased expression of VCAM-1 in bronchial mucosa and bronchoalveolar lavage (BAL) fluid from patients with allergy-induced asthma [73]. In the same paper, in vitro studies revealed that IL-4-induced VCAM-1 expression stimulates the transendothelial migration of eosinophils across airway endothelial cells [73]. Furthermore, they also demonstrated that VCAM-1, but not E-selectin or ICAM-1, is significantly increased in IL-4-positive asthma patients, compared with IL-4-negative asthma patients [73]. Hakansson et al. further reported that patients with allergy-induced asthma have higher levels of blood eosinophil infiltrates than healthy individuals [74]. Adhesion assays with eosinophil reveal that IL-4 increases VCAM-1 expression in airway endothelial cells and results in the increased adhesion of eosinophils to VCAM-1 on the cells, emphasizing that the adhesion of eosinophil with VCAM-1 may play a central role in the pathogenesis of asthma.

Several reports have suggested the importance of the interactions between VCAM-1 and α4β1 integrin in asthma [66,75,76,77]. Nakajima et al. demonstrated that blocking VCAM-1 via murine anti-VCAM-1 antibody (M/K-1) decreased the eosinophil infiltration in OVA-induced asthma mouse models [66]. Pretolani et al. demonstrated that aerosol OVA inhalation in guinea pigs increased their bronchial reactivity, which is accompanied by distinct eosinophil infiltration in the bronchopulmonary tissue and accumulation in the BAL fluid [75]. Under these conditions, treatment with an anti-α4β1 integrin monoclonal antibody (HP1/2) inhibited antigen-induced bronchial hyperreactivity and inhibited eosinophil infiltration and accumulation in the bronchial tubes [75]. Furthermore, Milne and Piper also observed the higher levels of eosinophils in BAL fluid in OVA mice than in controls, supporting the important role of α4β1 integrin and VCAM-1 in the recruitment of eosinophils to the inflamed lung during bronchial hyperresponsiveness [76]. Chin et al. further showed the role of the α4β1 integrin and VCAM-1 on leukocyte trafficking in the airways of mice with OVA-induced asthma treated with anti-α4 integrin antibodies (PS/2, Rl-2) and anti-VCAM-1 monoclonal MoAb (M/K-2.7) [77]. They showed that all three antibodies significantly inhibited the recruitment of eosinophils and lymphocytes into BAL fluid and decreased inflammation in lung tissue, suggesting that α4 integrin and VCAM-1 may have important roles in the recruitment of T cells and eosinophils in OVA-induced airway inflammation [77]. Recently, Lee et al. developed a novel human anti-VCAM-1 monoclonal antibody (HD101) that binds strongly to Ig-like domains 1 and 2 of human and mouse VCAM-1 [40]. They confirmed that the antibody can effectively ameliorate eosinophilic inflammation and airway hyperresponsiveness in the OVA mouse model [40]. Further, they also showed that the antibody inhibits the adhesion of U937, EoL-1, and CD4^+^ T cells to human VCAM-1 [40]. In addition, the adhesion of U937 and EoL-1 to TNFα-activated HUVECs also was blocked effectively by the antibody in a concentration-dependent manner [40]. Together, these results suggest that antibodies targeting VCAM-1 may be an effective therapeutic approach to alleviate asthma.

### 3.3. Transplant Rejection

Owing to mismatched organ donors and recipients, organs can be rejected by the innate and adaptive immune system of the recipient in allotransplantation or xenotransplantation [78,79]. Transplant rejection is initiated by the infiltration of leukocytes toward inflamed sites. Lymphocytes and monocytes are central players in this response, and ultimately cause graft damage [80,81]. Transplant rejection is a complex interplay between the recipient’s leukocytes and the donor’s endothelium.

In organ transplantation, the endothelium of the grafted organs is the first barrier between self and non-self that is encountered by host leukocytes [82]. Cell adhesion molecules, particularly VCAM-1, expressed on endothelium are closely related to leukocyte transmigration and recruitment toward inflammation sites. Over the past several decades, many studies of transplant rejection have demonstrated the upregulation of VCAM-1 expression on the endothelium of grafted organs, including the liver, kidneys, lungs, and heart. For example, in frozen sections of human liver allografts, VCAM-1 expression is significantly increased in the vascular and sinusoidal endothelium of the acutely rejected grafts [83]. Ultrastructural immunogold localization results show that VCAM-1 expression also is upregulated on the peritubular capillary endothelium in renal allograft rejection and is strongly focally expressed on the basolateral surface of tubules [84]. Furthermore, VCAM-1 mRNA levels progressively increase with rejection times in pulmonary endoarterial biopsy samples of canine lung allograft models [85]. Additionally, according to several studies, endomyocardial biopsies of acutely rejected human cardiac allografts showed increased VCAM-1 expression, which correlates with the degree of CD3+ T cell infiltrates and rejection [86,87,88]. Taken together, these data suggest that VCAM-1 expression may play an important role in transplant rejection, and could be a useful biomarker for transplant rejection.

Several reports show that the modulation of the interaction between VCAM-1 and α4β1 integrin may alleviate transplant rejection. In a skin allograft C3H/HEJ murine model, an anti-mouse VCAM-1 antibody (MK1.9), either alone or in combination with an anti-mouse VLA-4 antibody (PS/2) significantly improved the graft survival of C3H/HEJ mice [89]. Stegall et al. also reported that in an islet allograft CBA murine model, an anti-mouse VCAM-1 antibody (MK2.7) prolonged islet allograft survival by more than 100 days with an islet graft survival rate of 75% [90]. Furthermore, the treatment of cardiac allograft C57BL/6 mice with an anti-mouse VCAM-1 antibody (MK2.7) alleviated rejection and prolonged graft survival by approximately five days [87]. In addition, MK2.7 antibodies specifically bind to the Ig-like domains 1 and 4 of VCAM-1 [91]. Recently, we demonstrated that the anti-VCAM-1-D6 chimeric monoclonal antibody we developed can improve islet allograft survival [43]. In detail, treatment with the antibody prolonged graft survival for more than 110 days in an islet allograft C57BL/6 murine model [43]. Furthermore, we found that the antibody significantly blocks the migration of CD4^+^ T cells and macrophages toward the grafted islets [43]. Intriguingly, our in vitro results show that, in contrast with other antibody blockades targeting the interaction between VCAM-1 and α4β1 integrin, our antibody uniquely inhibits the transendothelial migration of leukocytes without affecting leukocyte adhesion [43]. Together, these results suggest that the Ig-like domain 6 of VCAM-1 plays an important role in leukocyte transmigration in transplant rejection.

## 4. Role of VCAM-1 in Cancer

### 4.1. VCAM-1 in Angiogenesis

Recently, increasing amounts of evidence have shown that VCAM-1 is closely associated with tumor angiogenesis and metastasis. In this section, we will highlight the specific roles of VCAM-1 on tumor angiogenesis and metastasis.

Angiogenesis is a physiological process in which new blood vessels form from pre-existing blood vessels. It is finely regulated by many pro-angiogenic factors, including vascular endothelial growth factor (VEGF), epidermal growth factor, angiopoietin, and hepatocyte growth factor, and anti-angiogenic factors containing thrombospondin, endostatin, and angiostatin [92,93,94,95,96,97].

Tumor angiogenesis is a hallmark of cancer [98,99], and VEGF is a central player for regulating tumor angiogenesis [100]. Currently, bevacizumab, a humanized monoclonal antibody targeting VEGF, is one of the best treatments for patients with various cancers, including breast, brain, lung, ovarian, and renal cancers [101]. However, bevacizumab also regulates many other cellular functions, such as the activation of the coagulation cascade, kidney function, blood pressure, vascular homeostasis, bone marrow function, and thyroid function [102]. Thus, long-term bevacizumab use can lead to various adverse effects, such as hypertension, proteinuria, bleeding, and gastrointestinal perforation [103,104]. Furthermore, long-term bevacizumab use also provokes drug resistance in cancer therapy [105,106]. In this regard, new therapeutic targets in angiogenesis need to be identified.

Many studies have shown the relevance of VCAM-1 in angiogenesis. Yong-Bin et al. reported that VCAM-1-positive tissue has a higher microvessel density than VCAM-1-negative tissue in gastric cancer [107]. Byrne et al. reported that serum VCAM-1 levels correlate with the microvessel density of breast cancer, suggesting that serum VCAM-1 may be a surrogate marker of angiogenesis in breast cancer [108]. Other studies show that VEGF can upregulate the expression of VCAM-1 expression on endothelial cells [109,110]. Much attention has been paid to the interaction between VCAM-1 and α4β1 integrin in angiogenesis. For example, Garmy-Susini et al. first observed that VCAM-1 and α4β1 integrin were individually expressed on vascular smooth muscle cells and endothelial cells in the developing vessels of breast cancer, and found that the administration of an anti-murine VCAM-1 antibody (M/K-2) specifically reduced microvessel formation in Matrigel plug mouse models [111]. Furthermore, another report shows that in vitro exposure to anti-VCAM-1 antibody blocked IL-4– and IL-13–induced tube formation, and angiogenesis induced in vivo by IL-4 and IL-13 was inhibited by an antibody against α4 integrin [112].

Recently, we identified the Ig-like domain 6 of VCAM-1 (VCAM-1-D6) as a potential angiogenic target [113]. Using siRNA-mediated VCAM-1 knockdown, we found that VCAM-1 downregulation reduces TNFα-induced HUVEC migration and tube formation [113]. Competition assay results showed that TNFα-induced HUVEC tube formation is specifically inhibited by VCAM-1-D6 fused with Fc but not by Fc alone, indicating that VCAM-1-D6 is a key domain in TNFα-induced angiogenesis [113]. Furthermore, we found that VCAM-1 overexpression in HEK293 cells increases VCAM-1–mediated cell–cell contacts, and that these interactions are inhibited by a rabbit/human chimeric monoclonal antibody that is specific to the VCAM-1-D6 that we developed [113]. Lastly, we demonstrated that the antibody can specifically inhibit not only TNFα-induced HUVEC migration and tube formation, but also TNFα-induced vessel sprouting from rat aorta without severe endothelial cell toxicity [113]. These data suggest that VCAM-1 may be a key target for modulating tumor angiogenesis.

### 4.2. VCAM-1 in Metastasis

Tumor metastasis is a complex process that involves the invasion and intravasation of tumor cells from primary sites to enter circulation via the lymph or blood system, extravasation of these circulating tumor cells into distant tissues, and tumor formation in distant organs. Despite the remarkable development of cancer therapeutics, metastasis is closely associated with high mortality rates in cancer patients [114,115].

VCAM-1 expression seems to be closely implicated in the metastasis of various cancer cells. For example, through a comparative gene profile analysis of parental MDA-MB-231 breast cancer cells and in vivo isolates exhibiting lung metastatic activity, Minn et al. found that VCAM-1 expression is upregulated in metastatic breast cancer cells to the lungs [116]. Liu et al. showed that VCAM-1 expression correlated with the clinicopathological grade of gliomas [117]. Furthermore, mesothelium VCAM-1 expression was negatively associated with progression-free and overall survival in patients with epithelium ovarian cancers [118]. VCAM-1 expression also is upregulated in lung cancers [119]. In addition, VCAM-1 is overexpressed in colorectal cancer, and is associated with lymph node metastasis, clinical stage, and tumor progression in patients with colorectal cancer [120].

Several VCAM-1 modulators further show the relationship of VCAM-1 in tumor metastasis. The in vitro transfection of microRNA-181a-5p, a tumor suppressor, downregulates VCAM-1 expression and impedes IL-17-induced proliferation and the migration of H226 and H460 non-small cell lung cancer cells [121]. The shRNA-mediated knockdown of VCAM-1 in MDA231 breast cancer cells reduced adhesion with U937 promonocytic leukocyte cells, and also inhibited lung metastasis [122]. The pretreatment of melanoma cells with an antibody specific to α4β1 integrin completely inhibited the IL-1-induced augmentation of lung colonies, suggesting the importance of VCAM-1–α4β1 integrin interaction in this process [123]. Another study also demonstrated that the pretreatment of B16–BL6 cells with either anti-α4β1 or anti-VCAM-1 antibodies (M/K-2) destroyed TNFα-enhanced pulmonary lung colonies [124].

In a previous study, we reported that VCAM-1 is highly overexpressed in lung cancer tissues, and that high VCAM-1 expression is closely associated with the poor survival of patients with lung cancer [119]. In this study, using the siRNA-mediated knockdown of VCAM-1, we identified VCAM-1 as a key molecule regulating the invasion of A549 lung cancer cells. Then, using phage display technology, we developed a novel human monoclonal antibody that is specific to human and mouse VCAM-1-D6 by isolating antibody clones from a human synthetic antibody library. We confirmed that this antibody specifically inhibited the invasion of A549 and NCI-H1299 lung cancer cell lines, suggesting that VCAM-1-D6 may be a novel potential therapeutic target in VCAM-1-mediated lung cancer invasion. Taken together, these results suggest that targeting VCAM-1 may be an effective strategy for regulating tumor metastasis.

## 5. Conclusions

**Figure 2:**
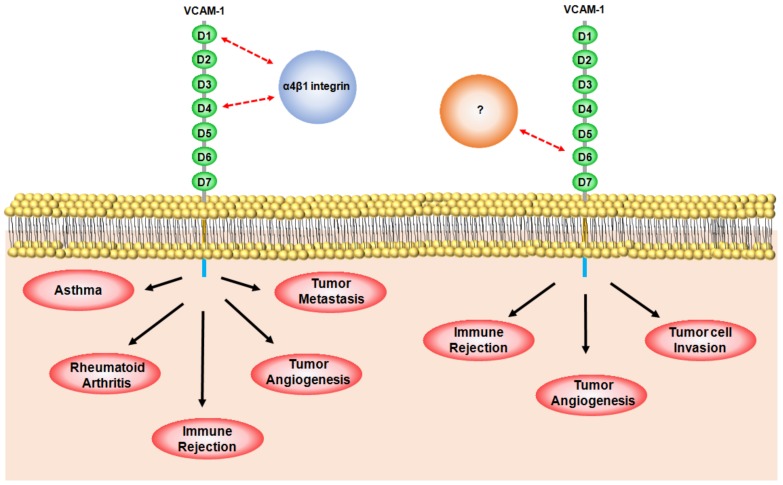
Schematic representation of the specific role of each vascular cell adhesion molecule-1 (VCAM-1) immunoglobulin (Ig)-like domain in immunological disorders and cancer. The direct interaction between Ig-like domain 1 (D1) and/or domain 4 (D4) of VCAM-1 on activated endothelial cells and α4β1 integrin (blue circle) on leukocytes is closely associated with asthma, rheumatoid arthritis, transplant rejection, tumor angiogenesis, and tumor metastasis; Ig-like domain 6 (D6) of VCAM-1 is important in transplant rejection, tumor angiogenesis, and tumor cell invasion. However, further research is necessary in order to identify the binding partners (orange circle) of D6 and their regulatory mechanisms.

VCAM-1 is a key cell adhesion molecule involved in inflammation that is closely implicated in various immunological disorders, including rheumatoid arthritis, asthma, transplant rejection, and cancer (Table 1). VCAM-1 is a potential therapeutic target in immunological disorders and cancer. The interaction of α4β1 integrin, a major binding partner of VCAM-1, with Ig-like domain 1 or 4 of VCAM-1 is critical for the progression of rheumatoid arthritis, asthma, transplant rejection, angiogenesis, and metastasis. Additionally, our recent studies also suggest that the Ig-like domain 6 of VCAM-1 is a potential therapeutic target in transplant rejection, angiogenesis, and tumor cell invasion (Figure 2). However, in order to understand the pathological mechanism of VCAM-1 in immunological disorders and cancer, further research is necessary to identify VCAM-1 domain-specific binding partners, and elucidate their regulatory mechanisms. Furthermore, the generation of interaction blockades that specifically inhibit the strong association between VCAM-1 and its binding partners has been very challenging. Although some mouse monoclonal antibodies have been used for elucidating the role of VCAM-1 in disease, we need to develop human or humanized antibodies that are specific to VCAM-1, less immunogenic, and have broad cross-species reactivity for preclinical and clinical studies. Luckily, the recent advent of recombinant antibody technology can overcome major hurdles for developing human antibodies that can be useful for either research or therapeutic use. Future studies with these specific blockades will create new avenues for better understanding the regulatory mechanisms of VCAM-1 as a potential therapeutic target in immunological disorders and cancer.

## Figures and Tables

**Table 1:** Relevance of VCAM-1 in immunological disorders and cancer. RA: rheumatoid arthritis.

Disease	Animal Model	Applied Antibody	Effect	Reference
RA	DBA/1 mouse model of collagen-induced arthritis	Anti-VCAM-1 monoclonal antibody (M/K-2.7)	Reduction in overall clinical severity of disease	Carter et al., 2001 [55]
	Chimeric SCID mouse/human synovial tissue model	Anti-VCAM-1 polyclonal antibody	Inhibition of marrow-derived endothelial progenitor cell adhesion to RA synovial tissue	Silverman et al., 2007 [59]
Asthma	BALB/c mouse model of ovalbumin-induced asthma	Anti-VCAM-1 monoclonal antibody (M/K-1)	Prevention of eosinophil and lymphocyte infiltration into the trachea	Nakajima et al., 1994 [66]
	C57BL/6 mouse model of ovalbumin-induced asthma	Anti-VCAM-1 monoclonal antibody (M/K-2.7)	Inhibition of eosinophil and lymphocyte recruitment into the bronchoalveolar lavage fluid	Chin et al., 1997 [73]
	BALB/c mouse model of ovalbumin-induced asthma	Anti-VCAM-1 monoclonal antibody (HD101)	Attenuation of macrophage, neutrophil, and eosinophil recruitment into bronchoalveolar lavage fluid	Lee et al., 2013 [40]
Immune rejection	C3H/HEJ murine model of skin allograft	Anti-VCAM-1 monoclonal antibody (MK1.9)	Prolongation of skin allograft survival	Gorcyznski et al., 1995 [85]
	CBA murine model of islet allograft	Anti-VCAM-1 monoclonal antibody (MK2.7)	Prolongation of islet allograft survival	Stegall et al., 2001 [86]
	C57BL/6 mouse model of cardiac allograft	Anti-VCAM-1 monoclonal antibody (M/K-2)	Prolongation of cardiac allograft survival	Pelletier et al., 1992 [83]
	C57BL/6 mouse model of islet allograft	Anti-VCAM-1 monoclonal antibody (MK2.7)	Prolongation of islet allograft survival	Lee et al., 2012 [43]
Cancer	Matrigel plug nude mouse model	Anti-VCAM-1 monoclonal antibody (M/K-2)	Inhibition of neovascularization	Garmy-Susini et al., 2005 [107]
	C57BL/6 mouse model of pulmonary metastasis	Anti-VCAM-1 monoclonal antibody (M/K-2)	Reduction of TNFα-enhanced pulmonary lung colonies	Okahara et al., 1994 [120]
